# The Role of Polymerase Chain Reaction of High-Risk Human Papilloma Virus in the Screening of High-Grade Squamous Intraepithelial Lesions in the Anal Mucosa of Human Immunodeficiency Virus-Positive Males Having Sex with Males

**DOI:** 10.1371/journal.pone.0123590

**Published:** 2015-04-07

**Authors:** Carmen Hidalgo-Tenorio, Mar Rivero-Rodriguez, Concepción Gil-Anguita, Javier Esquivias, Rodrigo López-Castro, Jessica Ramírez-Taboada, Mercedes López de Hierro, Miguel A. López-Ruiz, R. Javier Martínez, Juan P. Llaño

**Affiliations:** 1 Infectious Disease Unit, University Hospital *Virgen de las Nieves*, Granada, Spain; 2 Pathology Service, University Hospital *Virgen de las Nieves*, Granada, Spain; 3 Gastroenterology Service, University Hospital *Virgen de las Nieves*, Granada, Spain; Georgetown University, UNITED STATES

## Abstract

**Objectives:**

To evaluate the advantages of cytology and PCR of high-risk human papilloma virus (PCR HR-HPV) infection in biopsy-derived diagnosis of high-grade squamous intraepithelial lesions (HSIL = AIN2/AIN3) in HIV-positive men having sex with men (MSM).

**Methods:**

This is a single-centered study conducted between May 2010 and May 2014 in patients (n = 201, mean age 37 years) recruited from our outpatient clinic. Samples of anal canal mucosa were taken into liquid medium for PCR HPV analysis and for cytology. Anoscopy was performed for histology evaluation.

**Results:**

Anoscopy showed 33.8% were normal, 47.8% low-grade squamous intraepithelial lesions (LSIL), and 18.4% HSIL; 80.2% had HR-HPV. PCR of HR-HPV had greater sensitivity than did cytology (88.8% *vs*. 75.7%) in HSIL screening, with similar positive (PPV) and negative predictive value (NPV) of 20.3 *vs*. 22.9 and 89.7 *vs*. 88.1, respectively. Combining both tests increased the sensitivity and NPV of HSIL diagnosis to 100%. Correlation of cytology *vs*. histology was, generally, very low and PCR of HR-HPV *vs*. histology was non-existent (<0.2) or low (<0.4). Area under the receiver operating characteristics (AUROC) curve analysis of cytology and PCR HR-HPV for the diagnosis of HSIL was poor (<0.6). Multivariate regression analysis showed protective factors against HSIL were: viral suppression (OR: 0.312; 95%CI: 0.099-0.984), and/or syphilis infection (OR: 0.193; 95%CI: 0.045-0.827). HSIL risk was associated with HPV-68 genotype (OR: 20.1; 95%CI: 2.04-197.82).

**Conclusions:**

When cytology and PCR HR-HPV findings are normal, the diagnosis of pre-malignant HSIL can be reliably ruled-out in HIV-positive patients. HPV suppression with treatment protects against the appearance of HSIL.

## Introduction

The past few decades have produced an increase in the incidence of anal squamous cell carcinoma (ASCC) due to the increase in risk groups such as men having sex with men (MSM) and immune-depressed individuals especially those infected with HIV [1]. ASCC is one of the most frequent non-AIDS-defining malignancies in the HIV-infected population [2] and several studies confirm higher prevalence of ASCC in HIV individuals relative to those who are seronegative [3]. Of note among the factors implicated in the appearance of ASCC are chronic human papilloma virus (HPV) infection, tobacco use, female gender, immune-suppression, and belonging to the MSM group of individuals [4].

The mucosa of the anal canal has characteristics in common with the uterine cervix, among which is the high susceptibility to the oncogenic effect of HPV [5]. In studies conducted in HIV-positive women in whom the role of HPV oncogene genotypes had been investigated in relation to the appearance of HSIL of the cervix, colonization by multiple genotypes and low levels of CD4 were observed to be associated with the dysplasia [6]. These results are similar to those communicated regarding anal mucosa of HIV-positive MSM individuals [7]. However, the effect of antiretroviral (ARV) treatment on ASCC is debated [8–14], since data have been presented indicating that the rates of progression of pre-malignant lesions to carcinoma are increased in the anal mucosa, relative to the general population [15].

Cost-effective analysis of implementing a protocol of early diagnosis of dysplastic lesions of the anal mucosa in HIV-positive MSM patients was evaluated by an expert committee [16], and the outcomes were considered cost-effective. Despite this, the current guidelines for the treatment of HIV patients [17, 18, 19] have been very variable with respect to screening, diagnosis and follow-up of these lesions. Cytology is the technique that is currently employed for the screening of lesions in the anal mucosa. However, its diagnostic sensitivity is low, and its correspondence with the histology evaluation is variable, and often poor [20].

Hence, we conducted the current study to analyze the role of PCR of HR-HPV in the screening of HSIL in anal mucosa of HIV-positive MSM patients. The secondary objective was to identify the variables associated with the appearance of these lesions.

## Patients and Methods

This is a prospective cross-sectional study, conducted between May 2010 and May 2014, in a group of 201 HIV-positive MSM patients recruited consecutively into a program of screening, diagnosis, treatment and follow-up of dysplastic lesions of the anal mucosa. The HIV-positive patients were from among those receiving attention in the Infectious Disease Unit of the *Hospital Universitario Virgen de las Nieves* (Granada, Spain) the ethics committee of which approved the study.

The inclusion criteria were: adult (≥18 years of age) HIV-positive MSM patients. The exclusion criteria were: females, heterosexual HIV-positive males, and history of anal canal neoplasia at the time of recruitment into the study.

The objectives and conditions of the study were explained to the patients at the first clinical visit, and fully-informed written consent to participation in the study was obtained. Clinical, epidemiological and analytical data obtained during the study were codified for anonymity, according to the laws on protection of personal information currently in existence in Spain. All details of the methodology are contained within the manuscript. However, more information can be had from the corresponding author, if needed.

The clinical characteristics and laboratory work-up have been described previously [14].

At the clinical visit, 2 mucosa samples were taken from the anal canal with cotton swabs soaked in physiologic saline, and stored in liquid medium for the detection and genotyping of the HPV using the polymerase chain reaction (PCR) technique (GeneAmp PCR System 9700, Applied Biosystems, Roche), and for cytology using the ThinPrep Pap Test (Thin Prep Processor 2000, Hologic Corp). Both samples were sent to the anatomy-pathology laboratory where the same senior pathologist of the research team (JE) carried out the cytology evaluation, validation of PCR methodology, and histology analyses. HPV genotypes 16, 18, 26, 31, 33, 35, 39, 45, 51–53, 56, 58, 59, 66, 68, 73 and 82 were considered high-risk (HR-HPV). Genotypes 6, 11, 34, 40, 42–44, 54, 55, 57, 61, 70–72, 81, 83, 84 and 89 were considered low-risk (LR-HPV). The virus was classified on HPV-18 species as genotypes 39, 45, 59, 68; and of the HPV-16 species as genotypes 31, 33, 35, 52, 58, 67 [21].

Subsequently, anoscopy was performed by the gastroenterologist specialist of the research team (MLdeH), within an interval of between 4 and 12 weeks from the cytology evaluation. A standard endoscope of 9 mm with working channel of 2.8 mm was used, without any image enhancement. A short exploration of 15–20 cm was performed with retrovision maneuver to better visualize the pectinea line. Samples were taken for histology examination using an endoscopic retrograde cholangio-pancreatography (ERCP) catheter. Sampling was from all four quadrants (anterior, posterior, left and right) to access not only the lesions (acetolugol-negative zones) but also ostensibly-normal mucosa. This was to avoid false negatives in anoscopy without amplification system.

The cytology classification was that of Bethesda [22] which classifies the lesions into 3 types: atypical squamous cells (ASC), LSIL and HSIL.

The histology classification employed divides the lesions into LSIL (AIN1/condyloma), HSIL (AIN2, AIN3/Carcinoma *in situ*), and invasive carcinoma [23].

### Statistical analyses

#### Sample size

The sample size calculation was performed with the Ene 2.0 statistical software package. In the primary objectives of the study (Sen, Spe, PPV and NPP) to achieve a cytological sensitivity of 75% with a specificity of 6% in the estimation and using an asymptotic normal 95% bilateral distribution, it was necessary to recruit 201 subjects into the study. For risk factors associated with the appearance of HSIL (secondary objective of the study), with a precision of 8% using normal asymptotic bilateral confidence interval of 95% assuming that the prevalence of infection and of any grade of anal dysplasia is 70%, it would be necessary to include 126 subjects in the study. With an expected loss of 10%, the recruitment would be 140 patients.

#### Descriptive analyses

The general description of the principal variables included central tendency and dispersion (mean, standard deviation, median, percentiles) for the quantitative variables, and the absolute frequencies with 95% confidence intervals (95%CI) for the qualitative variables.

The prevalence and 95%CI were calculated for HPV, dysplasias obtained from cytology, and dysplasias obtained from the histology evaluation. Diagnostic success, not only from cytology but also from PCR of HR-HPV of dysplasias that included HSIL lesions, was defined by the receiver operating characteristics (ROC) curves. The results were considered poor: 0.5 to 0.6; acceptable: 0.6 to 0.75; good: 0.75 to 0.9; very good: 0.9 to 0.97; excellent 0.97 to 1. The degree of concordance between cytology, PCR of HR-HPV, and biopsy results was analyzed using the Kappa index. The results of the test were evaluated using the classification of Landis and Koch in which a value of k < 0.20 would be considered poor; 0.21 to 0.40 weak; 0.41 to 0.60 moderate; 0.61 to 0.80 good; 0.81 to 1.00 very good [24]. We analyzed the probability of HSIL in the histology evaluation as a function of the cytology and the PCR of HR-HPV independently, as well as in combination. Finally, we calculated sensitivity (Sen), specificity (Spe), positive predictive value (PPV) and negative predictive value (NPV) of the cytology and PCR of HR-HPV in the screening for HSIL.

#### Bivariate analyses

We used bivariate analyses to assess the relationship between the possible risk factors and the presence of HSIL (AIN2/AIN3). The Student *t*-test for independent samples was applied for quantitative variables that followed a normal distribution, while the Mann-Whitney test was employed for those variables that did not follow normal distributions. The Kolmogorov-Smirnov test was used to assess whether the different variables fulfilled the criteria of normal distribution. Comparison of differences between variables was with the Pearson χ^2^ test, or the Fisher exact test, as appropriate.

#### Multivariate analysis

Logistic multivariate regression was applied using the classic formula of Freeman [n = 10*(k+1)] [25]. Included in the model were the results that were statistically significant in the bivariate analyses, as well as those considered clinically relevant. Variables were individually introduced into the analyses manually, while leaving-out those variables that did not modify the model so that, finally, a model with those variables that did have a modifying effect was constructed. These included ARV treatment, current perianal condylomas, clinical history of syphilis, HPV 16, 18, 33, 61, 68, duration of HIV, duration of ARV treatment, number of HR-HPV genotypes in anal mucosa, CD4 nadir, VL <50 copies/mL, CD4 in the diagnosis of HIV, CD4, CD8 and VL in the course of the study. A method of selection using successive steps was employed such that, in each step, the probability of entry was 0.05 and that of exit was 0.10. The Hosmer-Lemeshow test was applied to assess the goodness of adjustment for the logistic regression model.

A value of p<0.05 was considered statistically significant in all the analyses. The statistical software used was the SPSS (version 15.0).

## Results

### Characteristics of the cohort of HIV-MSM patients

There were 201 patients included in the study. Mean age was 37 years. The CD4 nadir was 362 cells/μL, median time of clinical evolution of the HIV infection was around 36 months (interquartile range IQR: 15.3–106.8); 86.6% were on combination ARV therapy over a median period of 26.5 months, at which time there were 724.9 cells/μL CD4, and 71.1% had viral load (VL) <50 copies/mL. The characteristics are summarized in Table 1.

**Table 1:** General description of the patient study sample.

Variables	HIV-positive MSM n = 201
Age; years; mean ± SD	37.4 ± 9.5
Median number of anal partners in previous 12 months (IQR)	1 (1–5.8)
Use of condoms regularly; n (%); 95%CI	164 (81.6); 66–86
Education level	
Illiterate, n (%)	2 (1)
Primary school, n (%)	24 (11.9)
Secondary school, n (%)	64 (31.8)
University or equivalent, n (%)	111 (55.2)
Retired; n (%); 95%CI	13 (6.5); 1–9
Spanish national, n (%)	192 (95.5)
History of perianal and/or genital condylomatosis, n (%); 95%CI	69 (34.3); 28–41
Current perianal and/or genital condylomas, n (%); 95%CI	62 (30.8); 24–37
History of syphilis infection; n (%); 95%CI	42 (20.9); 12–32
Other STD; n (%); 95%CI	67 (33.3); 29–52
History of latent tuberculosis infection, n (%)	19 (9.6)
Median duration of HIV; months, IQR	36; 15.25–106.8
CD4 at time of HIV diagnosis, cells/μL; mean ± SD	473.08; ±344.4
CD4 nadir, cells/μL, mean; ± SD	362.7; ±248.5
CD4 current, cells/μL), mean; ± SD	724.9; ±603.9
CD8 current (cells/μL), mean; ± SD	1261.1; ±3465.3
VL current, log_10_n; mean ± SD	3.73; ±4.3
VL < 50 copies/mL, n (%); 95%CI	143 (71.1); 65–77
VL 50 and 199 copies/mL, n (%) 95%CI	13 (6.5); 3–10
VL 200 y 1000 copies/mL, n (%); 95%CI	9 (4.5); 2–7
VL > 1000 copies/mL, n (%); 95%CI	35 (17.4); 12–23
Viral relapse, n (%); 95%CI	11 (6.6); 3–10
AIDS stage (A3, B3, C), n (%), 95%CI	63 (31.3); 21–43
Treatment naïve, n (%)	27 (13.4)
Months of ARV treatment; median; IQR	26.5; 9–74.8
Chronic HCV infection, n (%); 95%CI	7 (3.5); 0–11
Chronic HBV infection, n (%); 95%CI	4 (2); 1–7
Smoking habit, n (%), 95%CI	93 (46.3); 34–58
EX-IVDA, n (%); 95%CI	2 (1); 1–7

HCV: hepatitis C virus infection; HBV: hepatitis B virus infection; EXIVDA: ex-intravenous drug abuser; STD: sexually transmitted disease; VL: HIV viral load; IQR: interquartile range; SD: standard deviation

### Results of the cytology, PCR of the HPV, and anal histology

In the anal cytology, we observed that only 38.4% of the patients had normal cytology, the rest having some grade of dysplasia; 51.1% had LSIL, 4.5% HSIL and 6.1% had ASCUS.

Also, 80.2% (95%CI: 65–85) had high-risk HPV genotype, 63.5% (95%CI: 50–73) low-risk genotype and 52.3% (95%CI: 33–56) both genotypes. The most-frequently isolated HPV genotypes in anal mucosa of low risk were: genotype 6 (17.3%), genotype 11 (14.7%) and genotype 61 (10.2%); and of high risk were: genotype 16 (31.5%), genotype 18 (14.7%), genotypes 42, 51 and 59 each at 14.2% and 55 and 68 each at 11.2%. Of the 201 anoscopies performed, only 33.8% had normal histology. The rest had some grade of dysplasia, of which 18.4% were HSIL (AIN2 or 3), and 47.8% LSIL (AIN 1) (Table 2).

**Table 2:** Outcomes of PCR of HPV, cytology and histology.

Outcomes of analyses	Study sample of HIV-positive MSM patients; n = 197
PCR of HPV, n (%); 95%CI	
HR-HPV	158 (80.2); 65–85
LR-HPV	125 (63.5); 50–73
HR- and LR-HPV	103 (52.3); 33–56
Median of HR-HPV	1 (1–3)
Median of LR-HPV	1 (0–2)
HPV-6	34 (17.3)
HPV-11	29 (14.7)
HPV-16	62 (31.5)
HPV-18	29 (14.7)
HPV-31	27 (13.7)
HPV-42	28 (14.2)
HPV-51	28 (14.2)
HPV-53	19 (9.6)
HPV-55	22 (11.2)
HPV-59	28 (14.2)
HPV-61	20 (10.2)
HPV-68	22 (11.2)
HPV 18 species (39,45,59,68)	83 (42.1); 29–59
HPV 16 species (31,33,35,52,58,67)	102 (51.8); 35–59
**Cytology**, n (%)	n = 198
Normal	76 (38.4)
LSIL	101 (51)
HSIL	9 (4.5)
ASCUS	12 (6.1)
**Anoscopy, Histology**, n (%)	n = 201
Normal	68 (33.8)
LSIL	96 (47.8)
HSIL	37 (18.4)

LSIL: low-grade squamous intraepithelial lesion; HSIL: high-grade squamous intraepithelial lesion; ASCUS: abnormality of uncertain significance; HPV: human papilloma virus; HR-HPV: high risk HPV; LR-HPV: low risk HPV; AIN: anal intraepithelial neoplasia.

### Value of cytology and PCR-HPV in the screening of HSIL

The sensitivity, specificity, PPV, and NPV of the anal cytology for the diagnosis of HSIL was 75.7% (95%CI: 0.62–0.9), 41.6% (95%CI: 0.34–0.49), 22.9% (95%CI: 0.15–0.3) and 88% (95%CI: 0.81–0.95), respectively. The corresponding values for PCR of HR-HPV were 88.8% (95%CI: 0.79–0.99), 21.8% (95%CI: 0.15–0.28), 20.3% (95%CI: 0.14–0.27) and 89.7% (95%CI: 0.8–0.99), respectively. Combining both tests (PCR HR-HPV and abnormal cytology) the corresponding values of Sen, Spe, PPV and NPV were 100%, 16.2%, 21.5% and 100%, respectively (Table 3).

**Table 3:** Comparison of anal cytology and PCR of HR-HPV in HSIL (AIN2/3) diagnosis.

	Sen (%)	Spe (%)	PPV(%)	NPV(%)	LR+ (95%CI); odds (%) (95%CI)	LR- (95%CI); odds (%) (95%CI)
Dysplastic cytology	75.7	41.6	22.9	88.1	1.30 (1.04–1.62); 23% (19–27%)	0.58 (0.32–1.06); 12% (7–20%)
PCR HR-HPV positive	88.8	21.8	20.3	89.7	1.14 (0.99–1.31); 20% (18–23%)	0.51 (0.19–1.34); 10% (4–23%)
Dysplastic cytology and/or HR-HPV positive	100	16.2	21.5	100	1.14 (0.99–1.31); 20% (18–23%)	0.5 (0.19–1.35); 10% (4–23%)
ASCUS	3	93.2	8.3	80.6	0.4 (0.05–2.97); 8% (1–41%)	1.04 (0.98–1.12); 19% (18–20%)
LSIL	59.5	50.9	21.8	50.9	1.21 (0.89–1.65); 22% (17–27%)	0.8 (0.52–1.21); 16% (11–22%)
HSIL	13.5	97.5	55.5	83	5.44 (1.53–19); 56% (26–81%)	0.89 (0.78–1.01); 17% (15–29%)

Sen: sensitivity; Spe: specificity; PPV: positive predictive value; NPV: negative predictive value; HR-HPV: high-risk human papilloma virus; LR+: positive likelihood ratio; LR-: negative likelihood ratio; CI: confidence interval; HSIL: high-grade squamous intraepithelial lesions

The probability of HSIL (AIN 2/3) diagnosis in the case of normal cytology was 11.8%; for dysplastic cytology was 22.9%; for PCR of HR-HPV was 20.3%. In the case of normal cytology and absence of HR-HPV, the probability of HSIL was zero.

Employing the Kappa Index (KI), we observed significant correlation between abnormal cytology and presence of LSIL (AIN1; KI: 0.19; p = 0.004) and HSIL (AIN2/3; KI: 0.09; p = 05) in anal histology, and strong negative correlation (i.e. disagreement) in case of normal cytology and normal histology (KI: -0.28; p = 0001) (Table 4).

**Table 4:** Correlations between cytology and PCR of HR-HPV of anal mucosa and histology.

	Normal histology n(%) Kappa; p	LSIL (AIN 1) n(%) Kappa; p	HSIL (AIN2/3) n(%) Kappa; p
Normal cytology (n = 76)	40(53.9) -0.28;0.0001	27(35.5) -0.22;0.04	9(11.8) -0.123;0.05
Abnormal cytology (n = 122)	25(20.5) 0.33; 0.0001	69(56.6) 0.19;0.004	28(22.9) 0.09; 0.05
LSIL (n = 101)	17(16.8) 0.33; 0.0001	62(61.4) 0.26;0.0001	22(21.8) 0.07; 0.25
HSIL (n = 9)	1(11.1) 0.03;0.16	3(33.3) -0.03;0.35	5(55.5) 0.16; 0.04
ASCUS (n = 12)	7(58.3) -0.05;0.05	4(33.3) -0.04;0.28	1(8.3) -0.56; 0.35
HR-HPV positive (n = 158)	43(27.2) 0.23; 0.001	82(51.2) 0.12;0.04	32 (20.3) 0.05;0.14
HR-HPV negative (n = 39)	22(56.4) 0.23; 0.001	13(33.3) 0.12;0.04	4(10.3) 0.046;0.1
Cytology normal & PCR HR-HPV positive (n = 50)	21(42) -0.07;0.12	20 40) -0.08; 0.16	9(18) -0.07;0.92
Cytology normal & PCR HR-HPV negative (n = 24)	18(75) -0.16;0.0001	6(25) -0.12;0.014	0(0) -0.17;0.01

HR-HPV: high risk-human papilloma virus; HSIL: high-grade squamous intraepithelial lesions.

Correlation of PCR HR-HPV and anal histology showed a borderline significant association with the presence of LSIL (AIN1; KI: 0.12; p = 0.04), and non-significant with HSIL (AIN2/3; KI: 0.05; p = 0.14) (Table 4).

**Fig 1:**
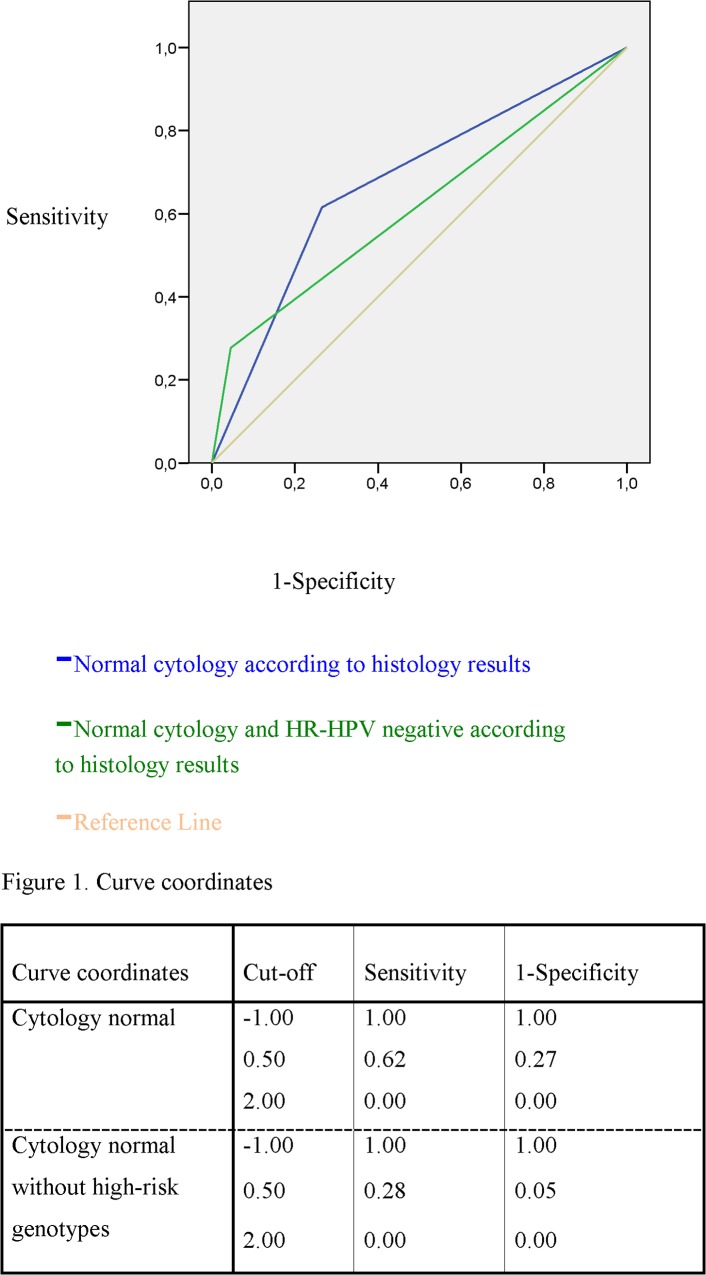
ROC curve of HR-HPV-negative and normal cytology *vs*. normal histology.

The degrees of concordance between the histology findings and the abnormal cytology and PCR HR-HPV were poor in both cases with an area under the receiver operating characteristics (AUROC) curve <0.5 (AUROC: 0.581; 95%CI: 0.482–0.681 and AUROC: 0.554; 95%CI: 0.455–0.653, respectively). In case of normal cytology and normal histology, AUROC curve was 0.67 (95%CI: 0.593–7.57) and, in the case of normal cytology without oncogenic genotypes, was 0.616 (95%CI: 0.528–0.704) i.e. in both cases there as good concordance (Fig 1).

### Risk factors associated with the appearance of HSIL

In the bivariate analysis of the different factors associated with the appearance of HSIL, risk variables observed were: replication of the HIV in plasma with a VL ≥1000 copies/mL (p = 0.029), the presence of genotype HPV-68 (p = 0.01), and HPV-61 associated with other oncogenic virus (p = 0.04). Protective factors observed were: receipt of ARV therapy (p = 0.001), viral suppression (VL <50 copies/mL; p = 0.01), and a longer time since HIV diagnosis (p = 0.001) (Table 5).

**Table 5:** Risk factors associated with the appearance of HSIL in anoscopy. Results of univariate analyses.

	HIV-positive MSM with HSIL n = 37	HIV-positive MSM without HSIL n = 164	p
Mean age, years; ± SD	35.5 ± 8.8	37.8 ± 9.7	0.17
Retired, n (%)	2 (0.05)	11 (0.07)	1
Anal partners in previous 12 months (IRQ)	1 (1–9.5)	1 (1–5)	0.47
Consistent condom use	28 (75.7)	136 (82.9)	0.3
Currently warts on penis and/or anus, n (%)	14 (37.8)	48 (29.3)	0.4
Previous history of:			
Genital and/or anal or perianal warts, n (%)	14 (37.8)	55 (33.5)	0.62
Syphilis, n (%)	5 (13.5)	37 (22.5)	0.22
ITL, n (%)	4 (10.8)	15 (9.2)	1
Smoking habit, n (%)	18 (48.6)	75 (45.7)	0.75
Months since HIV diagnosis, median (IQR)	18.5 (3–35.3)	46.5 (18.3–111.5)	<0.001
CD4 (cells/μL) at HIV diagnosis, mean (± SD)	549.5 (± 411.1)	454.9 (± 325.6)	0.15
CD4 nadir (cells/μL), mean (± SD)	380.3 (±302.6)	358.8 (±235.3)	0.64
CD4 (cells/μL), mean (± SD)	659.8 (±332.7)	739.7 (±649.6)	0.47
CD8 (cells/μL), mean (± SD)	994.9 (±411.5)	1321.6 (±3833.2)	0.6
VL log_10_n, mean (± SD)	4.07 (±4.52)	3.59 (±4.14)	0.16
VL < 50 copies/mL, n (%)	20 (54.1)	123 (75)	0.01
VL 50–199 copies/mL, n (%)	2 (5.4)	11 (6.5)	1
VL 200–1000 copies/mL, n (%)	2 (5.4)	7 (4.2)	1
VL > 1000 copies/mL, n (%)	11 (29.7)	24 (14.3)	0.029
Viral failure, n (%)	1 (3.8)	10 (7.2)	0.84
AIDS stage (A3, B3, C), n (%)	9 (24.3)	54 (32.9)	0.38
ARV treatment, n (%)	29 (78.4)	145 (88.4)	0.18
Months of ARV treatment, median (IRQ)	11.5 (1.25–27)	31 (11.25–83.75)	0.001
Current PCR of HPV			
LR-HPV, n (%)	25 (67.6)	100 (60.9)	0.41
HR-HPV, n (%)	32 (86.5)	126 (76.8)	0.15
LR & HR HPV, n (%)	20 (55.5)	83 (50.6)	0.66
HR-HPV, median (IQR)	2 (1–3.75)	1 (1–3)	0.13
LR-HPV, median (IQR)	1 (0–2)	1 (0–2)	0.57
Genotypes, n (%)			
HPV 18 species	19 (55.9)	64 (39)	0.15
HPV 16 species	20 (54.1)	82 (50)	0.62
HPV-16 & 18 simultaneously	5 (13.5)	7 (4.3)	0.046
HPV-6	9 (24.3)	25 (14.9)	0.17
HPV-11	9 (24.3)	20 (11.9)	0.054
HPV-16	15 (40.5)	47 (27.9)	0.15
HPV-18	8 (21.6)	21 (12.5)	0.16
HPV-33	0	16 (9)	0.047
HPV-39	6 (16.2)	13 (7.7)	0.12
HPV-42	6 (16.2)	22 (13.1)	0.64
HPV-45	3 (8.1)	19 (11.3)	0.77
HPV-51	7 (18.9)	21 (12.5)	0.32
HPV-52	3 (8.1)	16 (9.5)	1
HPV-53	7 (18.9)	12 (7.1)	0.054
HPV-53 & 16	4 (10.8)	6 (3.6)	0.16
HPV-55	1 (2.7)	21 (12.5)	0.09
HPV-59	6 (16.2)	22 (13.1)	0.64
HPV-61	8 (21.6)	12 (7.1)	0.01
HPV-61 with another HR-HPV	7 (18.9)	11 (6.5)	0.026
HPV-61 & 16	5 (13.5)	7 (4.3)	0.046
HPV-66	1 (0.59)	20 (54.1)	0.13
HPV-68	9 (24.3)	13 (7.7)	0.007
HPV-68 & 16	5 (13.5)	6 (3.6)	0.03

MSM HIV-positive: men who have sex with men and are HIV-positive; LTI: latent tuberculosis infection; HCV: hepatitis C virus; HBV: hepatitis B virus; HPV: human papilloma virus; EXIVDA: ex-intravenous drug abuser; VL: HIV viral load; HR-HPV: high-risk HPV; LR-HPV: low-risk HPV

Finally, in the logistic regression analysis evaluating the appearance of HSIL, the risk factors identified were: the presence of HPV genotype 68 (odds ratio OR: 20.1; 95%CI: 2.04–197.82), the prevalence of which was around 11.2%. The factors protective against the appearance of these lesions were: VL <50 copies/mL (OR: 0.312; 95%CI: 0.09–0.98), and having had a previous diagnosis of syphilis (OR: 0.193; 95%CI: 0.045–0.83).

## Discussion

In our cohort of HIV-positive MSM patients being screened for HSIL, we observed a higher sensitivity for PCR of HR-HPV than for cytology (88.8% *vs*. 75.7%), with low specificity in both cases (21.8 *vs*. 41.6%), and similar levels of PPV (20.3% *vs*. 22.9%) and NPV (89.7% *vs*. 88.1%). When combining both tests, the sensitivity and the NPV reached 100%. This would preclude the need for anoscopy in those patients with normal cytology and negative PCR HR-HPV. Our findings corroborate the results published previously by other authors proposing the advantage of adding PCR of HPV to the anal cytology in screening for HSIL lesions. Those published results, which are similar to ours, show a low specificity and low PPV but high sensitivity and NPV reaching 100% [26]. Other authors propose using PCR of HPV in anal mucosa in patients with cytology of uncertain significance [27, 28] in the HIV-negative MSM group [29] and, incidentally, to avoid under-diagnosis of HSIL which could be in the region of 22% [30]. The technique most employed in the screening of dysplastic anal mucosa lesions has been cytology; a test that is simple and cheap, the sample for which can be obtained by the clinician in the outpatient clinic, or even by the patient [31]. The sensitivity and specificity of anal cytology using Papanicolau in case of any grade of lesions varies between 47–93% and 32–50%, respectively. Cytology has a greater sensitivity in cases of patients with high-grade lesions, greater number of affected quadrants, HIV-positive and CD4 <200 cells/μL [32]. Our cohort of patients had excellent immune status, with a mean CD4 of 724 cells/μL and a prevalence of HSIL of 18%; both factors predictive of low sensitivity of cytology evaluation.

We encountered a low correlation between the anal cytology and histology (ƙ index <0.2), which we believe may be due to the low specificity of this technique, and which confers a low capacity to predict the histology grade of lesion in anal mucosa. This could be explained by the type of epithelium in the anus i.e. keratinized malpighian which would tend to obscure the lesions of the anal mucosa. Contrary to the mucosa of the cervix, the anal cytology would not be useful in grading the type of lesion. These results corroborate those of previous studies in which the cytology had low correlation with the histology e.g. approximately 40–50% of the ASCUs or LSIL lesions corresponding to precursor ASCC lesions [26, 33]. In our case, up to 22% of LSIL, 11.8% of normal and 8% of ASCUs corresponded histologically with HSIL.

Currently, there are authors who defend high-resolution anoscopy (HRA) as the ideal technique to screen for HSIL [34]. We, on the other hand, believe that HRA has several inconveniences. These include needing to be performed in a specialist center in which the apparatus is available, and with the appropriate clinical expertise together with the time and space available. Also, a possible complication, although slight, the procedure can cause discomfort to the patient e.g. rectal bleeding and pain during the sample taking. Sample acquisition for cytology is performed within about 20 seconds. It is not painful, without complications and, as well, can be performed by the patient. It does not require a specific site and can be taken into the same liquid buffer for the performance of the PCR of HPV and, as such, would avoid under-diagnosis of HSIL (AIN2 and 3) in patients with normal cytology, which in our case was 12%.

ASCC in HIV patients has special relevance due to its high incidence and, as well, being one of the non-AIDS defining neoplasias, most frequently identified in MSM and women with cervix pathology associated with HPV [35]. Also, its progression is greater [15], albeit the responses to chemo- and radio-therapy are similar to that in the seronegative population. Despite presenting with a protracted reduction in the CD4 levels following treatment, it does not produce an increase in the morbidity associated with HIV [36]. Hence, screening for pre-malignant lesions (HSIL) is of vital importance given that the treatment of these lesions could, according to the published studies, reduce the appearance of invasive ASCC; such treatments could range from topical medications (imiquimod, cidofovir), to ambulatory ablation of lesions using thermo-coagulation, or hospitalization for mucosectomy [37]. However, recommendations for the screening for precursors of ASCC vary considerably between the different scientific communities, and are even ambiguous [17, 18, 19]. For example the European Society (EACS) [17] recommends the screening in MSM annually or triennially using manual rectal examination and/or cytology (Papanicolau test). The American Society of Infectious Diseases recommends visual inspection together with manual rectal examination and, in HIV-positive MSM patients with history of condylomas, anal cytology is to be considered (albeit not as a standard technique). In the case of ambiguous/anomalous Papanicolau, high resolution anoscopy (HRA) is proposed [18]. Finally, the Spanish Group for the Study of AIDS (GESIDA) recommends, in non-specialized centers, a visual and manual examination. In the more specialized centers the recommendation is for an annual cytology examination for MSM, as well as for women with CIN2/3 or invasive carcinoma of the cervix. In the case of abnormal cytology, HRA is proposed [19].

With respect to HPV infection of the anal mucosa, we observed that 80.2% of our patients had high-risk genotypes, 63.5% had low-risk and 52.3% had both genotypes. The most frequent HPV genotypes isolated were those of low-risk HPV-6 (17.3%), HPV-11 (14.7%), and HPV-61 (10.2%); high-risk HPV-16 (31.5%), HPV-18 (14.7%), HPV-42, HPV-51 and HPV-59 (14.2% each), and HPV-55 and HPV-68 (11.2% each). This high prevalence of anal infection by high-risk and low-risk HPV has been communicated previously in HIV-positive MSM patients, and shown to be significantly associated with abnormal cytology [38].

In the multiple logistic regression analysis we observed, as a factor predictive of HSIL, the presence of the HPV-68 genotype (OR: 20.077; 95%CI: 2.04–197.82) and, as protectors, viral suppression of HIV(viral load <50 copies/μL; OR: 0.312; 95%CI: 0.099–0.984), and previous syphilis infection (OR: 0.193; 95%CI: 0.045–0.827). HIV viral suppression was related directly with the ARV treatment, which confers a protective effect against the appearance of HSIL in our study population of HIV-positive MSM patients. In the period prior to the introduction of ARV treatment (between the years 1985 and 1996) the incidence of ASCC in HIV-positive MSM patients was observed to be around 11 cases/100000 person-years. Since 1997, coinciding with the introduction of ARV treatment, there had been a 5-fold increase in the incidence relative to the previous incidence, with levels reaching around 55 cases/100000 person-years [9]. In the early period of ARV treatment (1997 to 2001) up to 134 and 144 cases/100000 person-years was observed [10]. The data from the NA-ACCORD cohort show an increase of this neoplasia in HIV patients coinciding with the early ARV treatment era; the levels of incidence remaining constant subsequently [11]. Conversely, the Swiss cohort in which the incidence of ASCC was measured, the finding was of a reduction of this neoplasia from around the “late ARV treatment” period (years 2002 to 2006) [12]. These data have been confirmed in other patient groups, including our own, in which this effect was measured in the late ARV treatment period [13, 14].

That previous infection with *Treponema pallidum* can be a protective factor against the appearance of HSIL could be interpreted as syphilis being a surrogate marker for early diagnosis of HIV and, thus, of the infection by HPV which is encountered in an earlier phase in which the appearance of the dysplastic lesions has not, as yet, occurred (14).

The most frequent genotype in our study population was HPV-16 with a prevalence of 31.5%. It was observed to be distributed homogenously between patients with and those without HSIL (40.5% *vs*. 27.9; p = 0.15). We assessed whether association with other high-risk viruses correlated with the appearance of HSIL. We observed that the combination with genotypes HPV-18 and HPV-68 was statistically significantly associated with HSIL (13.5 *vs*. 4.3; p = 0.04 and 13.5 *vs*. 3.6; p = 0.03, respectively). In published studies (not only in seropositive but also in seronegative) independently of the gender or sexual orientation, the genotype HPV-16 constitutes the most prevalent HR-HPV genotype in anal mucosa [39]

Genotype HPV-68, with a prevalence in our study population of 11.2%, was observed to be significantly associated with the appearance of HSIL (OR: 20; 95%CI: 2.04–197.8) such that the patients who presented with this genotype in the mucosa had a 20-fold higher risk of HSIL. This genotype has oncogenic capacity belonging to the C group of the phylogenetic tree of the papilloma virus, similar to genotypes 18, 39, 45 and 59 [14, 40]. These genotypes, as opposed to the HPV-16 in patients with similar immunological background, could more successfully avoid immune vigilance and give rise to dysplastic lesions in the anal mucosa [14, 41].

## Conclusions

In our cohort of HIV-positive MSM patients, PCR of HR-HPV was observed to have a greater sensitivity than cytology for the screening for HSIL. The correlation between cytology and the histology findings was non-significant, and may even provide contradictory results. In the absence of the oncogenic HPV virus, and with normal cytology, the probability of having HSIL approaches zero in our cohort of patients. In the group of HIV-positive MSM patients, based on the prevalence of HSIL, we recommend, irrespective of the center in which the patient is receiving attention, a systematic screening for precursor lesions of ASCC, at least with cytology. When the cytology is normal, we suggest conducting PCR of HPV. If the PCR-HPV findings are of high-risk genotypes, anoscopy should be performed. Anoscopy must be performed always in case of abnormal cytology. Finally, we need to highlight that the principal risk factor associated with HSIL was genotype HPV-68 and, as protector, viral suppression resulting from the administration of ARV therapy.

These findings warrant further investigation.
