# Correction: The Role of Polymerase Chain Reaction of High-Risk Human Papilloma Virus in the Screening of High-Grade Squamous Intraepithelial Lesions in the Anal Mucosa of Human Immunodeficiency Virus-Positive Males Having Sex with Males

**DOI:** 10.1371/journal.pone.0128165

**Published:** 2015-05-08

**Authors:** Carmen Hidalgo-Tenorio, Mar Rivero-Rodriguez, Concepción Gil-Anguita, Javier Esquivias, Rodrigo López-Castro, Jessica Ramírez-Taboada, Mercedes López de Hierro, Miguel A. López-Ruiz, R. Javier Martínez, Juan P. Llaño

In the Abstract, the final sentence in the Conclusions subsection is incorrect. The correct sentence should read: “HIV suppression with treatment protects against the appearance of HSIL.”
